# Maize *ZmLAZ1-3* gene negatively regulates drought tolerance in transgenic *Arabidopsis*

**DOI:** 10.1186/s12870-024-04923-x

**Published:** 2024-04-05

**Authors:** Haoqiang Yu, Bingliang Liu, Qinyu Yang, Qingqing Yang, Wanchen Li, Fengling Fu

**Affiliations:** 1 Maize Research Institute, Sichuan Agricultural University, Chengdu, 611130 People’s Republic of China; 2 College of Food and Biological Engineering, Chengdu University, Chengdu, 610106 People’s Republic of China

**Keywords:** Ectopic expression, Drought, Maize, ZmLAZ1-3

## Abstract

**Background:**

Molecular mechanisms in response to drought stress are important for the genetic improvement of maize. In our previous study, nine *ZmLAZ1* members were identified in the maize genome, but the function of *ZmLAZ1* was largely unknown.

**Results:**

The *ZmLAZ1-3* gene was cloned from B73, and its drought-tolerant function was elucidated by expression analysis in transgenic *Arabidopsis*. The expression of *ZmLAZ1-3* was upregulated by drought stress in different maize inbred lines. The driving activity of the *ZmLAZ1-3* promoter was induced by drought stress and related to the abiotic stress-responsive elements such as MYB, MBS, and MYC. The results of subcellular localization indicated that the ZmLAZ1-3 protein localized on the plasma membrane and chloroplast. The ectopic expression of the *ZmLAZ1-3* gene in *Arabidopsis* significantly reduced germination ratio and root length, decreased biomass, and relative water content, but increased relative electrical conductivity and malondialdehyde content under drought stress. Moreover, transcriptomics analysis showed that the differentially expressed genes between the transgenic lines and wild-type were mainly associated with response to abiotic stress and biotic stimulus, and related to pathways of hormone signal transduction, phenylpropanoid biosynthesis, mitogen-activated protein kinase signaling, and plant-pathogen interaction.

**Conclusion:**

The study suggests that the *ZmLAZ1-3* gene is a negative regulator in regulating drought tolerance and can be used to improve maize drought tolerance via its silencing or knockout.

**Supplementary Information:**

The online version contains supplementary material available at 10.1186/s12870-024-04923-x.

## Background

Maize originates in the tropical hot and humid regions of South America. It has a high consumption of water and is highly sensitive to drought stress, which is one of the major environmental constraints of maize production [1–3]. Plants adapt to drought stress by stomatal aperture, cuticle thickening, root development, osmoregulation, life cycle shortening, reactive oxygen species (ROS) removal, and many other morphological or physiological responses through complex signaling cascades [4–8]. In the progress of conventional breeding, maize improvement for drought tolerance is not significant although a lot of efforts have been paid for over a hundred years [1]. In recent years, more and more efforts have been adopted in biotechnological improvements, such as genetic engineering through the overexpression or mutation of drought-responsive genes, as well as fast-developing genomics-assisted breeding, transcriptomics, proteomics, genome editing, and high-throughput phenotyping [1, 9–13]. However, all these attempts heavily depend on the detailed elucidation of the physiological mechanisms and molecular pathways that mediate the response of maize to drought stress [1, 3, 14].

Lazarus 1 (LAZ1) is a transmembrane protein with a conserved domain of DUF300 in eukaryotes, which functions as an organic solute transport protein in vertebrates [15, 16]. The first discovery of the LAZ1 protein in plants was from the *acd11* (*accelerated cell death 11*) mutant of *Arabidopsis*, and its function was described as causing autoimmune cell death and leading to pathogen-triggered hypersensitivity reactions [17–19]. In *Arabidopsis*, two LAZ1 proteins (LAZ1 and LAZ1H1) were shown to localize on the vacuolar membrane and play an important role in maintaining vacuolar integrity through brassinosteroid (BR) signal transduction [20].

In our previous studies, nine members of the ZmLAZ1 family were identified from the maize genome and clustered into three subfamilies together with their orthologs in *Arabidopsis* and rice. Although the members of the same subfamily shared similar structures of predicted proteins, their expression patterns in different organs and developmental stages, responses to abscisic acid (ABA) induction, simulated drought, and high salt stress, as well as physicochemical properties, signal peptides, and transport substrates, were diverse from each other [21]. Under the regulation of the ZmBES1/BZR1-11 transcription factor, ZmLAZ1-4 transports zinc ions on the membranes of the cytoplasm, chloroplast, and vacuole, and regulates zinc homeostasis in maize [22]. The expression of the *ZmLAZ1-3* gene in the roots and shoots of model inbred line B73 was significantly upregulated by drought stress [21]. Hence, in the present study, the *ZmLAZ1-3* gene was cloned from B73, and its drought-tolerant function was elucidated by expression analysis in different inbred lines and promoter activity detection under simulative drought stress, subcellular localization, and phenotyping and transcriptomics analysis of transgenic *Arabidopsis*.

## Materials and methods

### Expression analysis

The RNA-sequencing (RNA-seq) data of the *ZmLAZ1-3* gene in different maize organs and development stages were downloaded from the MaizeGDB database (https://www.maizegdb.org/) [23], and used for tissue-specific expression analysis. The seeds of maize genotypes 200B, 81,565, B73, Zheng58, ZNC442, SCML0849, and Dan340 with different drought tolerance were obtained from Sichuan Agricultural University and were cultivated in Hoagland nutrient solution [24–26]. As described in our previous studies [27, 28], the seeds were germinated on filter paper for 48 h, transferred into Hoglandʹs solution, and cultured in a greenhouse with 16 h light and 8 h dark at 28 °C. After two weeks, 60 uniform seedlings of each line were divided into three biological replicates and treated with 16% polyethylene glycol 6000 (PEG-6000) for 0 (control), 6, 12, 24, and 48 h. The roots and shoots were sampled separately, frozen in liquid nitrogen, and used for total RNA extraction by using RNAiso plus kit (TaKaRa, Dalian). After removing possible genomic DNA contamination by using DNase (TaKaRa, Dalian), each RNA sample was quantified using NanoDrop™ OneC (Thermo Scientific, USA) and reverse transcribed into cDNA by using the PrimeScript™ reagent kit (TaKaRa, Dalian). The quantitative real-time PCR (qRT-PCR) was performed TransScript® II Two-Step RT‒PCR SuperMix (TransGen, China) and a Bio-Rad CFX96™ Real-Time PCR system according to the methods described in our previous study [27, 28]. A 283 bp fragment of the *ZmLAZ1-3* gene was amplified using primer pair F1/R1 (Table S1) with three technical replicates. Meanwhile, a 135 bp fragment of the *ZmEF1α* gene was amplified using primer pair F2/R2 (Table S1) and used as an internal control [29]. The relative expression levels were calculated with the comparative 2^−ΔΔCT^ method [30] and the statistical significance was analyzed by using the IBM-SPSS software v.12.0 (http://www-01.ibm.com/software/analytics/spss/).

### Promoter activity assay

According to the sequence cloned and sequenced by Liu et al. [21]. , *cis*-affecting elements were predicted from the genomic DNA sequence 2000 bp upstream of the CDS of gene *ZmLAZ1-3* by using PlantCARE software (http://bioinformatics.psb.ugent.be/webtools/plantcare/html/). Genomic DNA was extracted from seedlings of mode inbred line B73 by CTAB method, and used for amplification of the full-length (2000 bp) promoter *pZmLAZ1-3*, as well as its 1126 bp 5’-terminal deleted promoter *pZmLAZ1-3* (1126 bp) missing the MYB and MBS elements at − 1939 and − 1898, and − 520 bp 5’-terminal deleted promoter *pZmLAZ1-3* (520 bp) missing the MYC, MYC, MYB, and MYB elements at − 1099, − 918, − 706, and − 524 bp, by using high fidelity Phanta Max Super-Fidelity DNA Polymerase (Vazyme Biotech, Nanjing) and primer pairs Fp2000/Rp, Fp1126/Rp, and Fp520/Rp (Table S1), respectively. The amplified products were separated by agarose gel electrophoresis, purified by using Universal DNA Purification Kit (Tiangen, Beijing), sequenced at Tsingke Biotech (Beijing), and recombined into expression vector pCAMBIA-1305.1 between restriction sites *Hind* III and *Nco* I, respectively. As described by Lu et al. [31]. , each of the recombined vectors was transformed into *Agrobacterium* GV3101 and used to infiltrate leaves of *Nicotiana benthamiana* cultivated in a greenhouse at Sichuan Agricultural University. Three of the infiltrated leaves were incubated in 16% PEG-6000 for 72 h, while the other three were incubated in water (control) for the same time. Leaf discs were punched and stained with GUS staining solution. After being photographed, the leaf discs were used to assay GUS activity as described by Jefferson et al. [32].

### Subcellular localization

The CDS of *ZmLAZ1-3* without stop code was amplified from the above cDNA sample of B73 by using high fidelity Phanta Max Super-Fidelity DNA Polymerase (Vazyme Biotech, Nanjing) and primers F3/R3 (Table S1), and recombined into expression vector pCAMBIA2300-*35 S-eGFP* restriction sites *Kpn* I and *Sal* I. The recombined vector pCAMBIA2300-*35 S-ZmLAZ1-3-eGFP*, as well as the blank vector pCAMBIA2300-*35 S-eGFP*, was transformed into *Agrobacterium* GV3101 and used to infiltrate leaves of *N*. *benthamiana*, respectively, according to the methods of Lu et al. [31]. The infiltrated leaves were incubated at room temperature for 3 days, and monitored for green fluorescence of the eGFP and spontaneous fluorescence of chlorophyll under a laser confocal microscope (LSM 800, ZIESS) at excitation wavelengths of 488 and 580 nm, respectively. The constructed vector pCAMBIA2300-*35 S-ZmLAZ1-3-eGFP*, together with the plasma membrane marker gene (*OsRAC3*) vector pm-*OsRAC3-mCherry* [22, 33], was precipitated onto gold particles (60 μm) and used to bombard the fifth scales of onion bulbs in helium biolistic gun (BioRad, USA), respectively. After incubating at 28 °C under dark for 24 h, the bombarded onion scales were monitored for green fluorescence of eGFP and fluorescence of OsRAC3-mCherry the same as above.

### Transformation and phenotyping of ***Arabidopsis***

The *Agrobacterium* strain transformed by vector pCAMBIA2300-*35 S-ZmLAZ1-3-eGFP* above was used to transform wild-type (WT) *Arabidopsis* (Col-0) obtained from The Arabidopsis Information Resource (TAIR) using the method of floral dip. Transgenic lines were screened by 50 mg/L kanamycin on 1/2 MS plates, identified by PCR amplification of *ZmLAZ1-3*, used for harvesting seeds individually. In T_3_ generation, the homozygous lines without segregation on 1/2 MS plates containing 50 mg/L kanamycin were selected and used for next study. The ectopic expression of *ZmLAZ1-3* was identified by observation of the eGFP fluorescence in the root tips and reverse transcription PCR (RT-PCR) of the cDNA synthesized from the total RNA extracted from the T_3_ lines by using *AtActin2* as an internal control.

The homozygous T_3_ lines identified by RT-PCR and WT were planted on 1/2 MS plates containing 0 (control), 250, and 300 mmol/L mannitol with six sample sizes and two replicates. After vernalizing at 4 °C for 2 days and cultured horizontally and vertically under optimal conditions for one week, the ratios of germination and root length were investigated and photographed, respectively. The other vernalized seeds of each line were germinated on 1/2 MS plates and transplanted into sterilized nutrient soil with three replicates. After being cultured under optimal conditions for two weeks, drought stress was conducted by stopping watering. After another two weeks, the drought-tolerant phenotypes were observed and photographed. Then, nine plants were sampled from each replicate for investigation of biomass, and then three for measurement of relative water content (RWC), relative electrical conductivity (REC), and malondialdehyde (MDA) content, respectively, as described by Ding et al. [34], Gaxiola et al. [35], and Yu et al. [36], respectively.

### Transcriptomics analysis of transgenic ***Arabidopsis***

The entire seedlings of each replicate were rinsed with PBS buffer, rapidly frozen in liquid nitrogen, packed in dry ice, and sent to Personalbio (Shanghai) for RNA extraction, enrichment of mRNA, construction of cDNA library, and sequencing on Illumina HiSeq3000 platform with pair-end 150. The FASTP software (https://github.com/OpenGene/fastp) was used to filter for clean reads by removing the sequencing adapter, and low-quality reads with unknown bases of more than 1% or Q ≦ 15 bases of more than 50% from the raw reads [37]. The clean reads were aligned to the *Arabidopsis* genome (https://ftp.ensemblgenomes.ebi.ac.uk/pub/plants/release-56/fasta/arabidopsis_thaliana/dna/Arabidopsis_thaliana.TAIR10.dna.toplevel.fa.gz) by using HISAT2 (http://www.ccb.jhu.edu/software/hisat/index.shtml [38]. The reads per gene were counted by using the HTSeq software (https://github.com/simon-anders/htseq) [39]. The differentially expressed genes (DEGs) between the homozygous T_3_ lines and WT were identified by using the DESeq software (https://learn.gencore.bio.nyu.edu/rna-seq-analysis/deseq/) with |log_2_(fold change)| ≥1 and *p*-value ≤ 0.05 [40], annotated for functions by using Gene Ontology (http://geneontology.org/), and enriched for mechanism pathways by using KEGG (https://www.kegg.jp/).

## Results

### Expression in response to drought stress

**Fig. 1:**
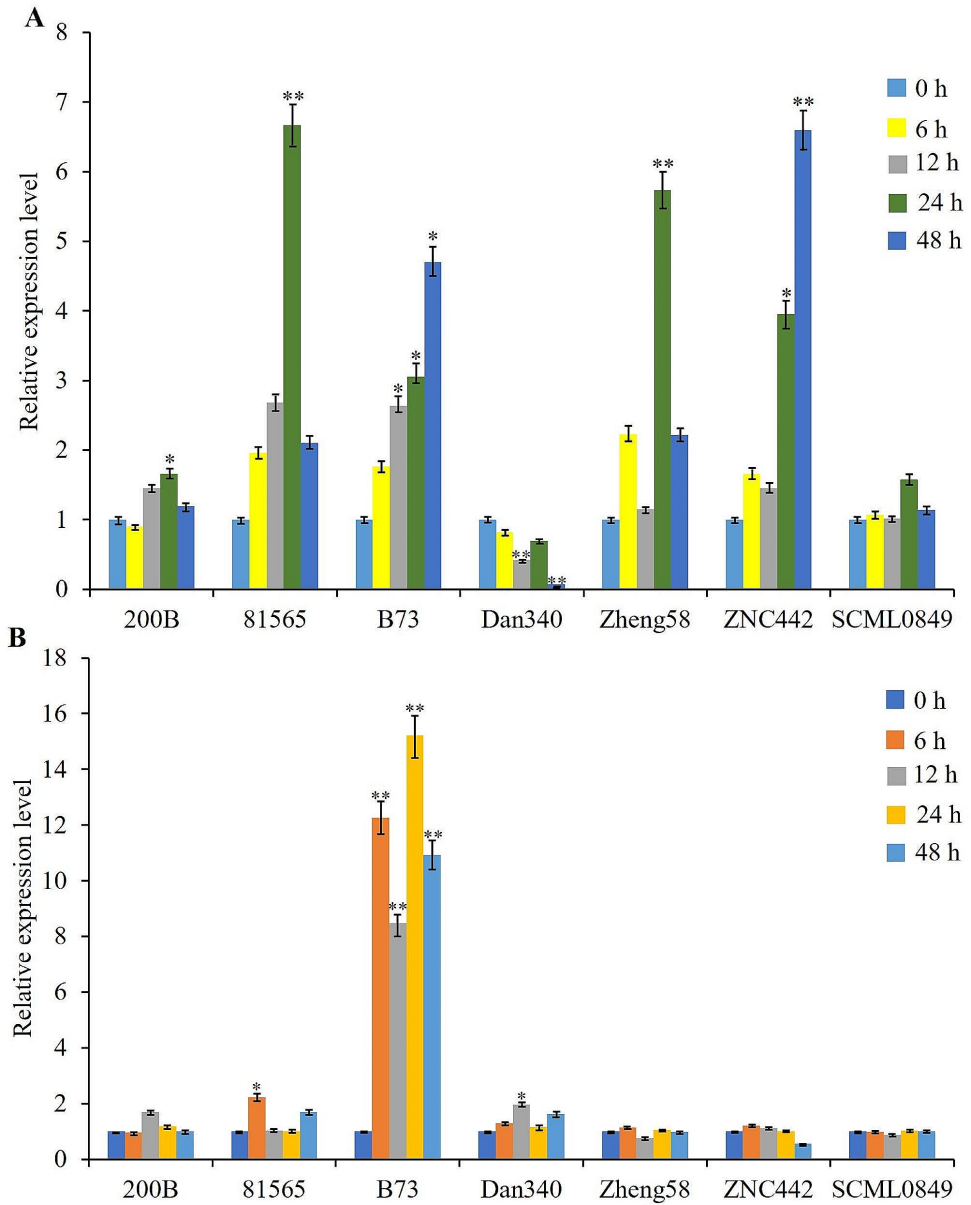
Relative expression levels of *ZmLAZ1-3* in the root (**A**) and shoot (**B**) of different inbred lines in response to simulative drought stress. Two-week-old maize seedlings were treated with 16% PEG-6000 for 0, 6, 12, 24, and 48 h. The roots and shoots were sampled separately. * and ** indicate significance at *p* < 0.05 and < 0.01, respectively

Analysis of RNA-seq data indicated that *ZmLAZ1-3* kept high-level expression in almost all organs and development stages without significant differentiation (Fig. S1). To explore the response of *ZmLAZ1-3* to drought stress, the RT-qPCR was performed to analyze the responsive expression in different maize genotypes. The results showed that its relative expression levels in the root of inbred lines 200B, 81,565, B73, Zheng58, and ZNC442 were significantly upregulated about five times of the control from 12 to 48 h of the simulative drought stress. In the root of inbred line Dan340, its relative expression level was extremely significantly downregulated about one time of the control from 12 to 48 h of the simulative drought stress. In the root of inbred line SCML0849, its relative expression level was non-significant with the control (Fig. 1A). Its relative expression level in the shoot of inbred lines B73 was extremely significantly upregulated about 11, 7, 13, and 10 times of the control from 6 to 48 h of the simulative drought stress, and of inbred lines 81,565 and Dan340 significantly upregulated about 1 and 0.5 times from 6 to 12 h. In the shoot of the other four inbred lines, its relative expression levels were non-significant with the control (Fig. 1B). The results confirm that the *ZmLAZ1-3* gene responds to drought stress and may function in regulating drought tolerance.

### ***ZmLAZ1-3*** promoter activity was enhanced by drought stress

**Fig. 2:**
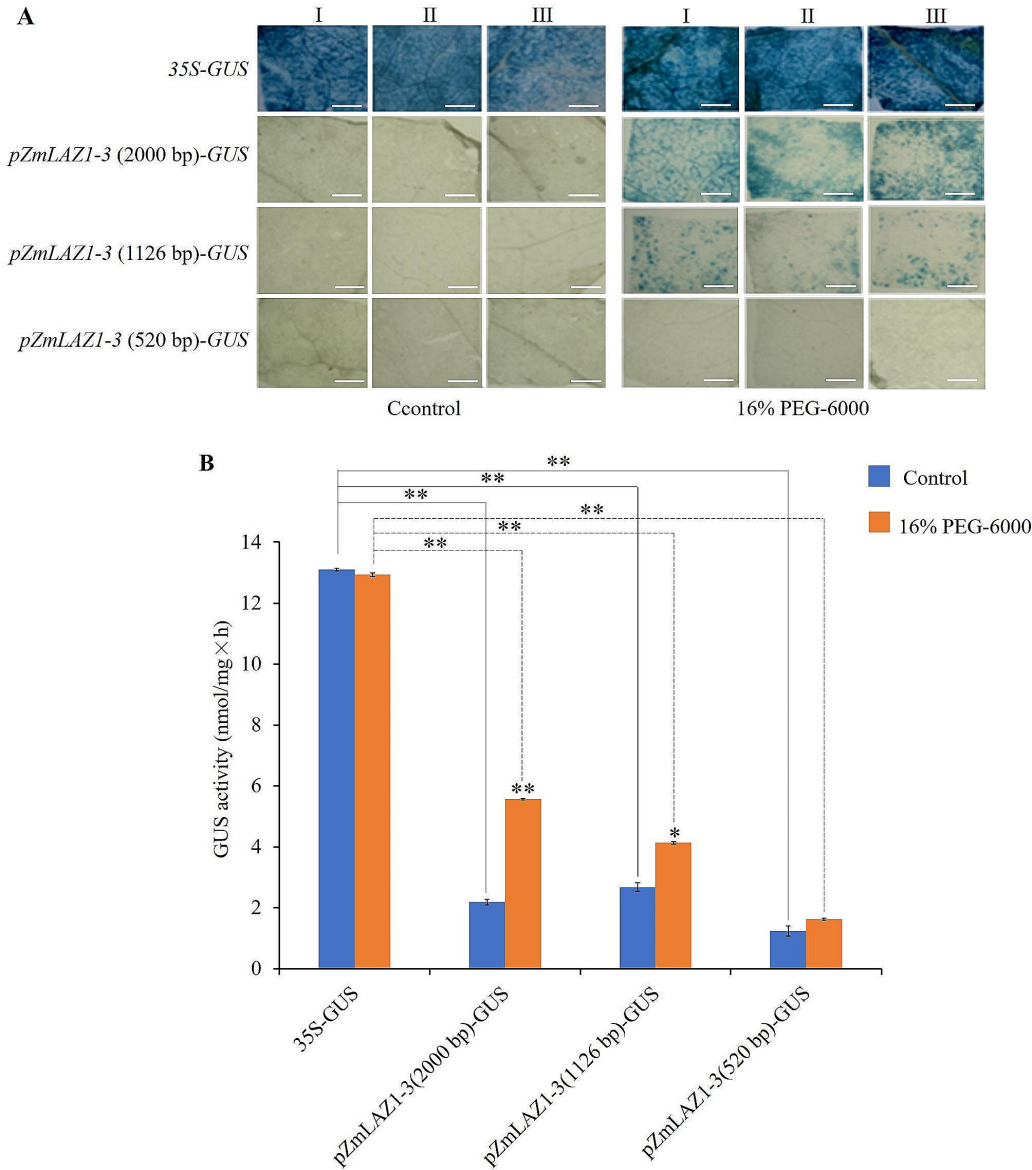
GUS staining (**A**) and activity (**B**) of tobacco leaf transiently expressing *GUS* gene under the control of promoter *pZmLAZ1-3* and its 5’-terminal deleted sequences missing different *cis*-acting elements. I, II and III represent three replicates. * and ** indicate significance at *p* < 0.05 and < 0.01, respectively. Scale bar is 1 cm

Dozens of *cis*-acting elements related to drought response, such as ARE, MYB, MYC, and MBS, as well as core elements of transcription initiation, such as CAAT-box, TATA-box, and A-box, were predicted by PlantCARE (Fig. S2). The GUS staining results showed that the tobacco leaf discs infiltrated with the positive control vector pCAMBIA-1305.1-*35 S-GUS* showed a dark blue color after incubating in both water and 16% PEG-6000. The leaf discs infiltrated with pCAMBIA1305.1-*pZmLAZ1-3* (2000 bp)-*GUS* and pCAMBIA1305.1-*pZmLAZ1-3* (1126 bp)-*GUS* showed extremely light blue spots after incubated in water and differently light blue spots after incubated in 16% PEG-6000. The leaf discs infiltrated with pCAMBIA1305.1-*pZmLAZ1-3* (520 bp)-*GUS* showed no blue spot at all after incubated in both water and 16% PEG-6000 (Fig. 2A). The GUS enzyme activity of the leaf discs infiltrated with the positive control vector pCAMBIA-1305.1-*35 S-GUS* was non-significantly different between incubated in 16% PEG-6000 and water (Fig. 2B), indicating the constitutive activity of promoter *35 S*. The GUS enzyme activity of the leaf discs infiltrated with pCAMBIA1305.1-*pZmLAZ1-3* (2000 bp)-*GUS* and pCAMBIA1305.1-*pZmLAZ1-3* (1126 bp)-*GUS* was extremely significantly or significantly higher after incubated in 16% PEG-6000 than that after incubated in water (Fig. 2B). These results indicated that the activity of promoter *pZmLAZ1-3* was upregulated in response to the simulative drought stress and the responsive effects of *cis*-acting elements MYB, MBS, and MYC.

### Subcellular localization

**Fig. 3:**
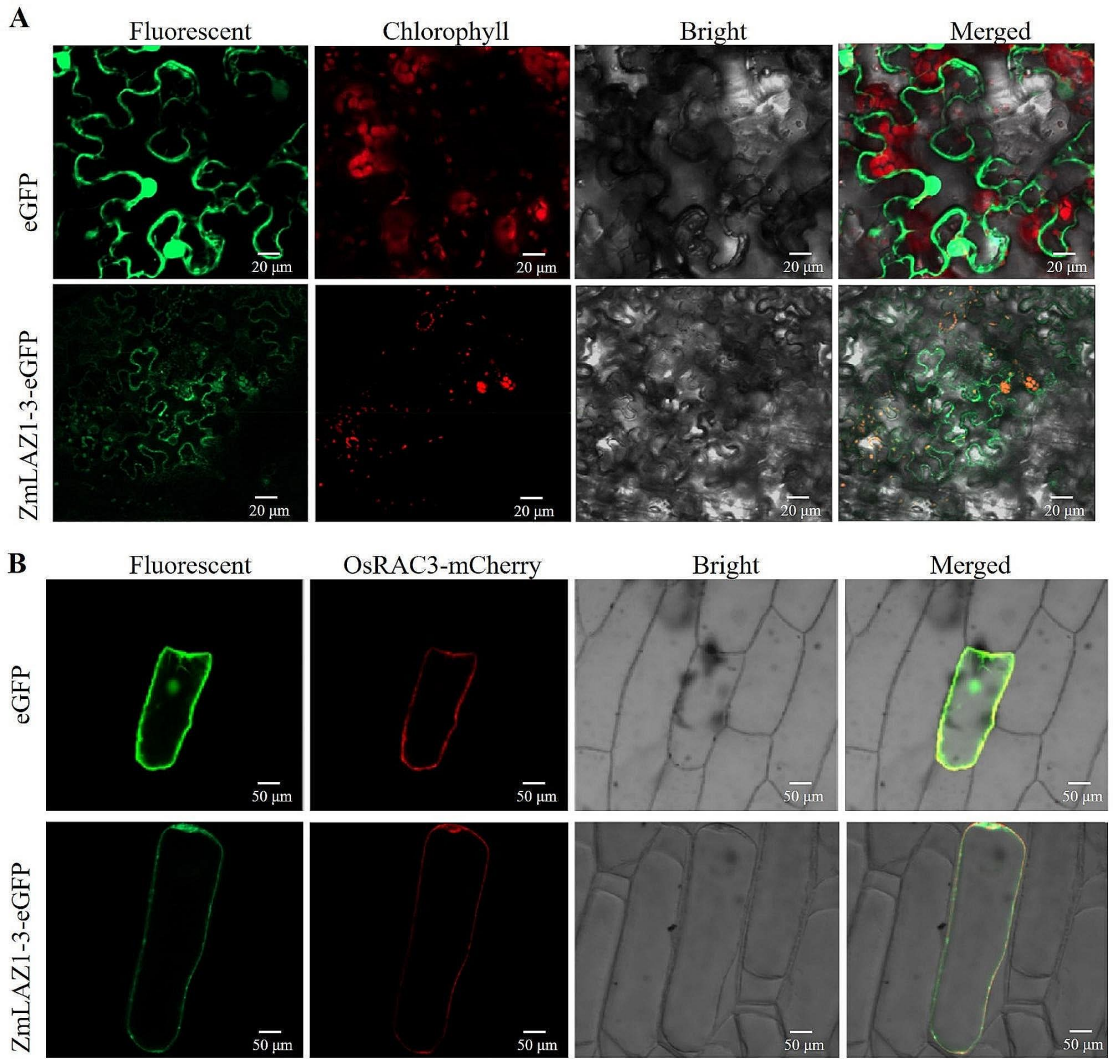
(**A**) Subcellular localization of ZmLAZ1-3 protein in tobacco mesophyll cell. (**B**) Co-localization of ZmLAZ1-3 and plasma membrane marker OsRAC3 in the epidermal cell of the onion bulb

At 488 nm of excitation wavelength, the green fluorescence was monitored on the plasma membrane and nucleus of the mesophyll cells infiltrated with the blank vector pCAMBIA2300-*35 S-eGFP*. In the mesophyll cells infiltrated with the recombined vector pCAMBIA2300-*35 S-ZmLAZ1-3-eGFP*, the green fluorescence was only monitored on the plasma membrane. At 580 nm of excitation wavelength, the spontaneous fluorescence was monitored on the chlorophyll of the mesophyll cells infiltrated with the blank vectors. In the mesophyll cells infiltrated with the recombined vector pCAMBIA2300-*35 S-ZmLAZ1-3-eGFP*, the green fluorescence completely matched the spontaneous fluorescence of chlorophyll (Fig. 3A), indicating the intracellular localization of the ZmLAZ1-3 protein on both the plasma membrane and chloroplasts.

At 488 nm of excitation wavelength, the green fluorescence was monitored on the plasma membrane and nucleus of the epidermal cells of onion bulbs co-transformed by the blank vector pCAMBIA2300-*35 S-eGFP* and the plasma membrane marker gene (*OsRAC3*) vector pm-*OsRAC3-mCherry*. At 580 nm of excitation wavelength, the fluorescence of the mCherry fused with the plasma membrane marker gene *OsRAC3* was monitored on the plasma membrane. In the epidermal cells of onion bulbs co-transformed by the recombined vector pCAMBIA2300-*35 S-ZmLAZ1-3-eGFP* and the plasma membrane marker gene (*OsRAC3*) vector pm-*OsRAC3-mCherry*, the green fluorescence was only monitored on the plasma membrane and completely matched the fluorescence of the mCherry fused with the plasma membrane marker gene *OsRAC3* (Fig. 3B), also indicating the intracellular localization of the ZmLAZ1-3 protein on the plasma membrane. The spontaneous fluorescence of chlorophyll could not be monitored because the chloroplasts did not develop in the epidermal cells of onion bulbs.

### Expression of ***ZmLAZ1-3*** enhanced drought sensitivity in transgenic ***Arabidopsis***

**Fig. 4:**
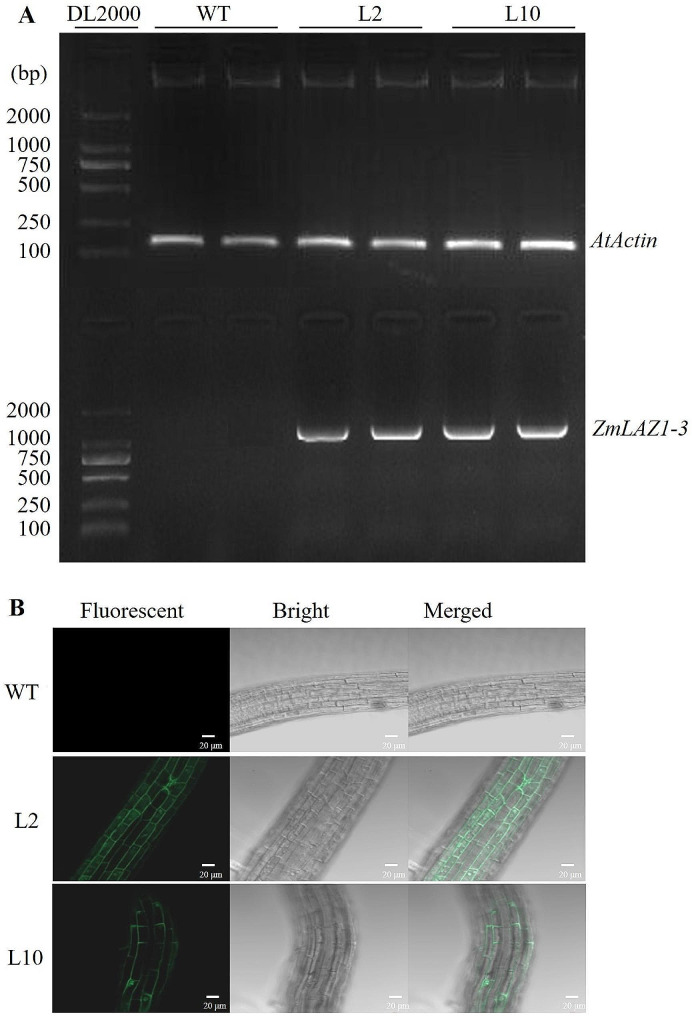
Ectopic expression of *ZmLAZ1-3* in the homozygous T_3_ lines identified by RT-PCR (**A**) and fluorescence monitoring of root tips (**B**). L2 and L10 indicate independent transgenic lines. WT means wild type and is used as control

After successive screening by 50 mg/L kanamycin on 1/2 MS plate and PCR identification of the transformed *ZmLAZ1-3* gene, two homozygous T_3_ lines were obtained. The ectopic expression of the transformed *ZmLAZ1-3* gene was identified by RT-PCR and fluorescence monitoring of their root tips (Fig. 4). The green fluorescence was monitored in the plasma membrane, which was consistent with subcellular localization (Fig. 4B).

**Fig. 5:**
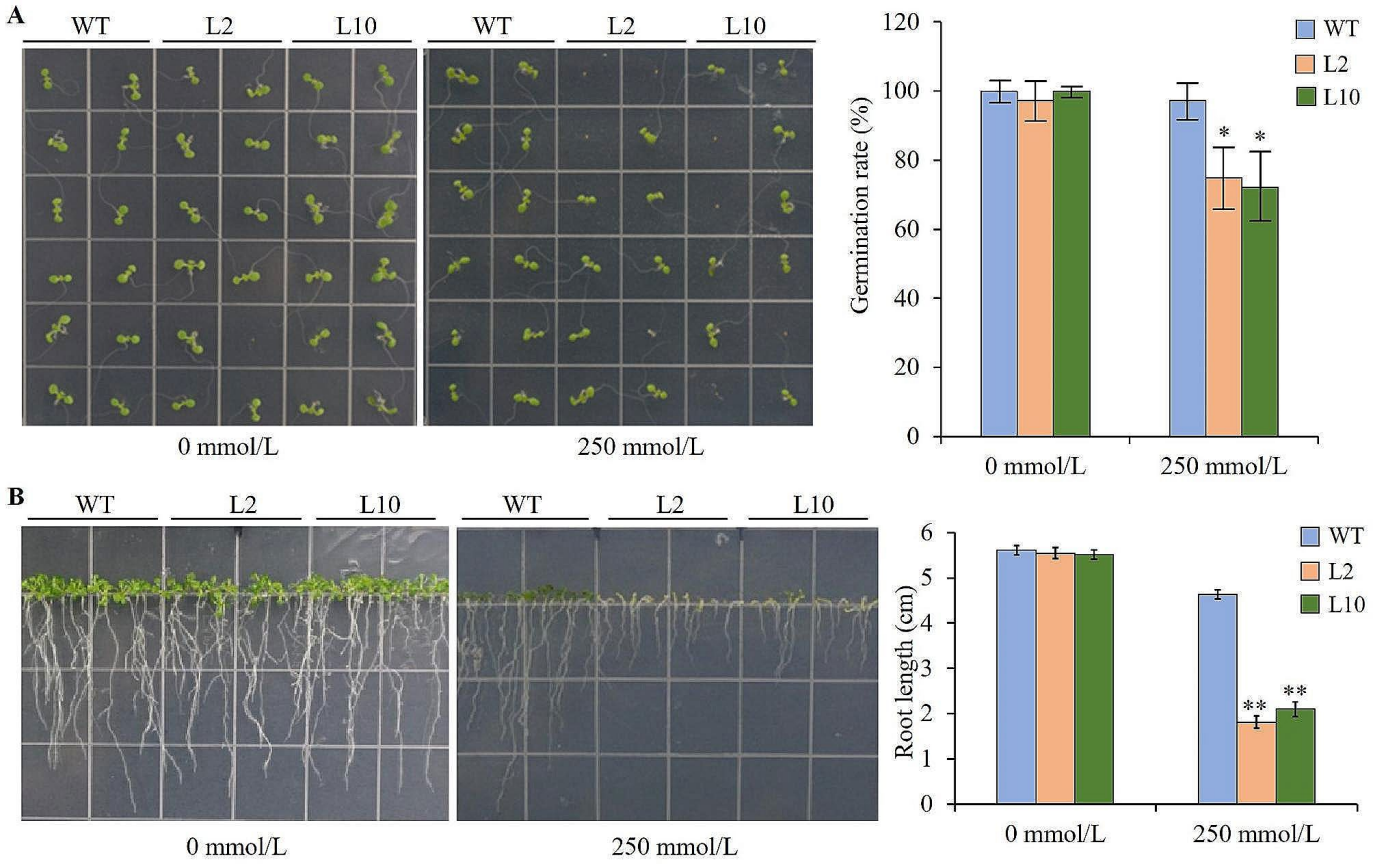
Germination rates (**A**) and root length (**B**) of the T_3_ lines under osmotic stress of 250 mmol/L mannitol. L2 and L10 are independent transgenic lines. WT is wild type. * and ** indicate significance at *p* < 0.05 and < 0.01, respectively

**Fig. 6:**
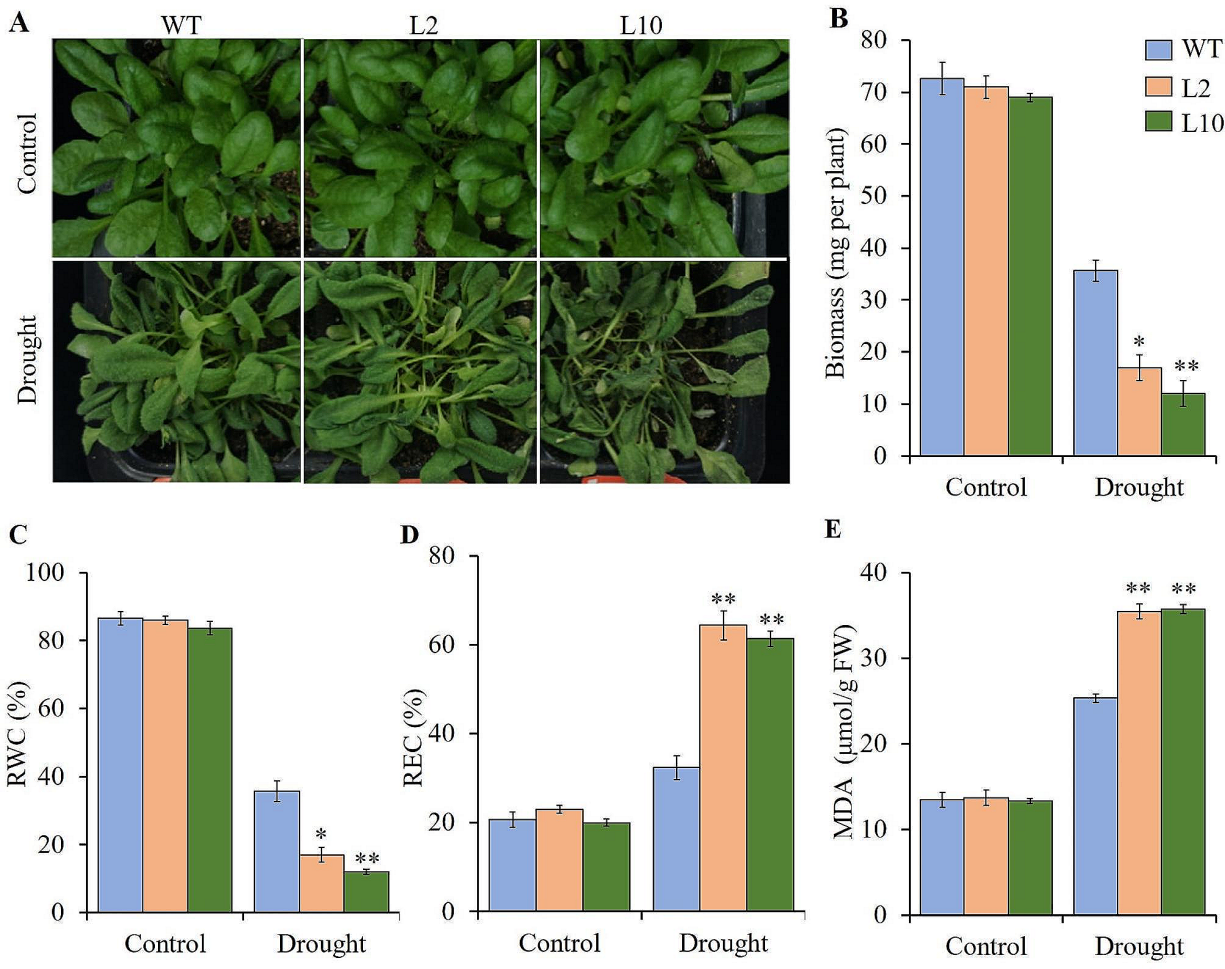
Phenotype of transgenic lines under drought stress. (**A**) Phenotype of each line. (**B**) Biomass of single plant. (**C**) RWC, relative water content. (**D**) REC, relative electrical conductivity. (**E**) MDA, malondialdehyde content. L2 and L10 are independent transgenic lines. WT is wild type. * and ** indicate significance at *p* < 0.05 and < 0.01, respectively

On 1/2 MS plates without mannitol, the germination rates and root length of the two T_3_ lines were non-significantly different compared to WT. Under osmotic stress of 250 mmol/L mannitol, their germination rates and root length were significantly lower or shorter than that of WT (Fig. 5). After two-week of natural drought, the two T_3_ lines showed obvious wilting, while WT remained normal (Fig. 6A). Moreover, the biomass of the two T_3_ lines was significantly or extremely significantly lower than that of WT (Fig. 6B). Before drought stress, the REL, REC, and MDA content of the two T_3_ lines were non-significantly different from control. After drought stress, these three drought-tolerance-related indicators of the two T_3_ lines were extremely significantly different from the control (Fig. 6C, D, E). All these results indicated that the ectopic expression of maize gene *ZmLAZ1-3* negatively regulates drought tolerance of *Arabidopsis*.

### ***ZmLAZ1-3*** regulated expression of stress-related genes in transgenic ***Arabidopsis***

**Fig. 7:**
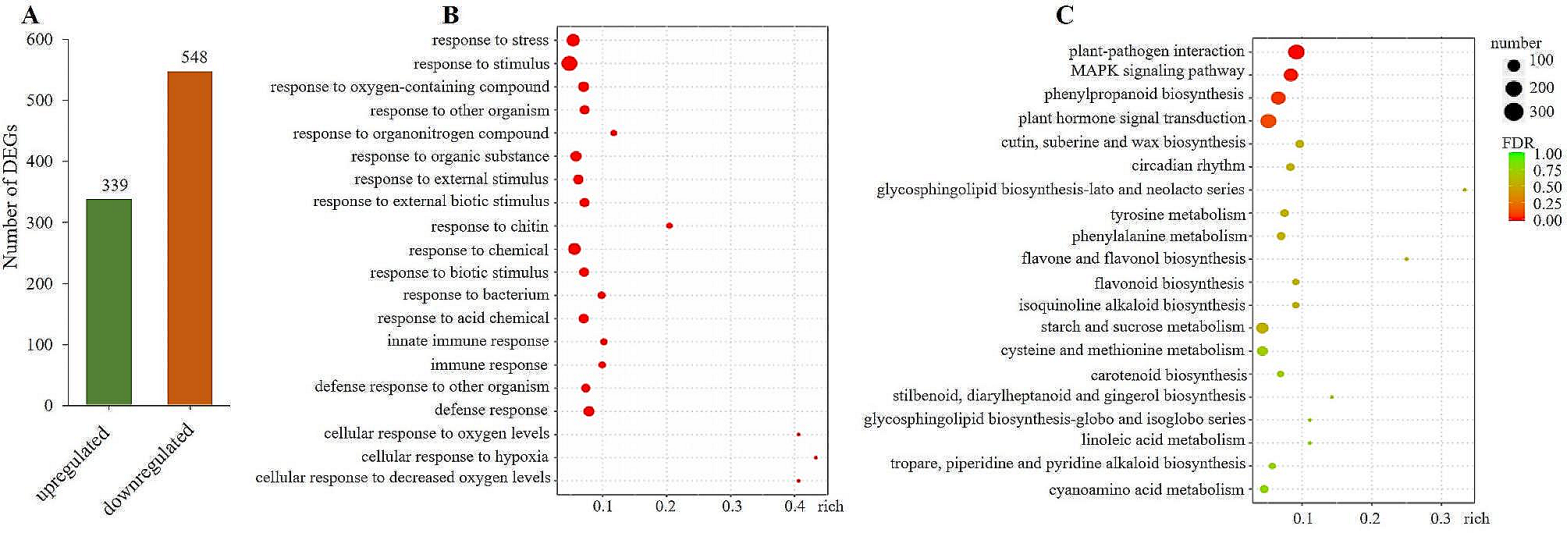
The differentially expressed genes (DEGs) in transgenic lines compared to WT. (**A**) The number of DEGs. (**B**) GO enrichment of DEGs. (**C**) KEGG pathway enrichment of DEGs

After quality control analysis of raw reads, each sample’s average Q20 and Q30 values were higher than 97% and 93%, respectively. The average base error rate of reads was 2.145‰. The mapping ratio of the clean reads against the *Arabidopsis* genome was more than 97%. Therefore, the dataset of RNA-seq could be used for the subsequent analysis. A total of 887 DEGs were identified from transgenic lines compared to WT, including 548 downregulated DEGs (Fig. 7A and Table S2). Among them, 14 *WRKY*, 12 *ERF*, 4 *DREB*, 5 *nanc*, 3 *NAC* genes were significantly inhibited in transgenic lines (Table 1). The results of the GO analysis showed that these DEGs are mainly associated with responses to abiotic stress (Fig. 7B). The results of KEGG enrichment showed that these DEGs were related to pathways of hormone signal transduction, phenylpropanoid biosynthesis, mitogen-activated protein kinase (MAPK) signaling, and plant-pathogen interaction (Fig. 7C).

**Table 1:** The downregulated genes encoding transcription factors in transgenic lines

Gene id	Gene Name	log2 Fold Change	P-value	Description
AT4G01250	*WRKY22*	-1.191507594	1.08733^E − 19^	Members of the WRKY transcription factor family
AT5G22570	*WRKY38*	-1.541626039	4.34194^E − 51^	
AT1G80840	*WRKY40*	-1.584783494	8.6472^E − 128^	
AT4G11070	*WRKY41*	-1.976777492	0.012521688	
AT2G46400	*WRKY46*	-1.791439223	1.24264^E − 73^	
AT5G64810	*WRKY51*	-1.954863755	1.07652^E − 31^	
AT4G23810	*WRKY53*	-1.290622598	1.56682^E − 61^	
AT2G40750	*WRKY54*	-1.60567954	5.43636^E − 43^	
AT3G01080	*WRKY58*	-1.351795125	9.30377^E − 06^	
AT2G21900	*WRKY59*	-2.045959275	0.010098014	
AT5G01900	*WRKY62*	-1.652154517	6.62775^E − 11^	
AT1G66600	*WRKY63*	-1.155533465	0.00018435	
AT1G80590	*WRKY66*	-1.836012452	0.001362193	
AT3G56400	*WRKY70*	-1.086906559	6.2048^E − 149^	
AT3G23240	*ERF1B*	-1.161995015	3.37348^E − 10^	ERF subfamily of the AP2 transcription factors
AT2G44840	*ERF13*	-1.257798343	1.29728^E − 23^	
AT1G28370	*ERF11*	-1.418384864	5.20604^E − 88^	
AT4G34410	*ERF109*	-2.941404287	0.000226089	
AT5G52020	*ERF025*	-1.685329116	7.38197^E − 20^	
AT1G33760	*ERF022*	-2.325362209	1.07275^E − 05^	
AT1G71520	*ERF020*	-1.172376969	0.009185143	
AT1G74930	*ERF018*	-1.538358794	1.3191^E − 134^	
AT1G19210	*ERF017*	-1.690066017	1.49547^E − 94^	
AT5G21960	*ERF016*	-1.767083414	2.70665^E − 15^	
AT1G77640	*ERF013*	-1.490089201	2.03291^E − 12^	
AT3G50260	*ERF011*	-1.014934868	3.14035^E − 15^	
AT1G12610	*DREB1F*	-1.851387404	5.5191^E − 16^	DREB subfamily of the AP2 transcription factors
AT1G63030	*DREB1E*	-3.620953363	0.047290184	
AT4G25470	*DREB1C*	-1.636181112	4.71973^E − 61^	
AT4G25490	*DREB1B*	-1.087772174	4.65022^E − 11^	
AT2G17040	*anac036*	-1.316296861	3.73501^E − 64^	NAC domain containing protein, subfamily of the NAC transcription factors
AT3G44350	*anac061*	-2.45712727	7.36919^E − 13^	
AT4G17980	*anac071*	-1.294854591	0.007426945	
AT4G28530	*anac074*	-1.099303726	0.000286744	
AT5G56620	*anac099*	-1.056837796	0.048976763	
AT1G61110	*NAC025*	-3.611559154	0.002372229	NAC transcription factor members
AT2G46770	*NAC043*	-3.254297455	0.012148486	
AT5G22380	*NAC090*	-2.074066324	2.12657^E − 06^	

## Discussion

The members of the LAZ1 protein family are identified because of their conserved domains of DUF300. Their physical and chemical properties, secondary structures, transmembrane structures, signal peptides, transport substrates, subcellular localization, and expression regulation of coding genes are greatly diverse [15, 20–22]. For example, ZmLAZ1-3 is the closet to ZmLAZ1-4 in the phylogenetic tree and conserved domains but does not have the functions of the latter, such as regulation of zinc homeostasis in maize by uptake of zinc from the soil and transport bi-directionally across the chloroplast and vacuole membrane [22].

Usually, the gene expression pattern can reveal its potential roles and is driven by its promoter, which possesses different *cis*-acting elements bonded by other factors [41, 42]. The expression of *ZmLAZ1-3* in maize root and shoot was upregulated 450 and 120 times in response to drought stress, respectively [21], while the expression of *ZmLAZ1-4* gene was downregulated in response to zinc and regulation of transcription factor ZmBES1/BZR1-11 of brassinolide (BR) signaling [22]. In the present study, the expression of *ZmLAZ1-3* in root and shoot was significantly upregulated several times in most inbred lines in response to drought stress and exhibited differences in different maize inbred lines (Fig. 1). This is possibly due to drought-responsive elements such as MYB, MBS, and MYC in its promoter sequence and potential genetic-variation in different maize genotypes (Fig. 2) [43, 44]. Previous studies found that genetic variation in the *ZmVPP1* and *TaNAC071* promoter, containing three and two MYB *cis*-elements, confers drought-inducible expression of *ZmVPP1* and *TaNAC071* and drought tolerance in maize and wheat, respectively [45, 46]. The SNP in an MYB cis-element in the *bsr-d1* promoter can enhance disease resistance in rice [47]. The MBS element in the *ZmSO* promoter region is responsible for ABA and drought-stress-induced expression [48]. Meanwhile, variation in the *ZmNAC080308* 5’-UTR region regulates gene expression and responds to drought stress [49]. The non-synonymous variants in *ZmSRO1d* also modulate drought resistance in maize [50].

Compared to WT, the transgenic lines with the *ZmLAZ1-3* gene showed obvious wilting stress, lower germination rates, root length, biomass, and RWC, and higher REC and MDA content (Figs. 5 and 6). All the above results indicated that the ectopic expression of *ZmLAZ1-3* reduced the drought tolerance of transgenic *Arabidopsis*. The ZmLAZ1-3 protein was localized on both plasma and chloroplast membranes (Fig. 3), speculating its function for maintaining the integrity of these membranes, since seven transmembrane structures were predicted by bioinformatics analysis, possible specific substrates were excluded [22]. A previous study suggests that LAZ1 and its paralogs can maintain the integrity of the vacuolar membrane in response to BR signaling [20]. The RWC, REC, and MDA content are widely used as biomarkers to evaluate plant tolerance to abiotic stresses due to stress-induced accumulation of reactive oxygen species (ROS) in cells resulting in damage of cell membrane and producing malondialdehyde [51–54]. In the present study, the expression of *ZmLAZ1-3* was upregulated in response to drought stress but negatively regulates drought tolerance (Figs. 1, 5 and 6). It likewise found that *VvWRKY18*, *TaSNAC4-3D*, *IbBBX28*, *ZmSAG39*, and *FtMYB11* genes from *Vitis vinifera*, *Triticum aestivum*, *Ipomoea batatas*, *Zea mays*, *Fagopyrum tataricum* are induced by drought stress but negatively regulate drought tolerance, respectively [55–59]. Moreover, many transgenic practices have proved that ectopic expression of exogenous genes under the control of strong constitutive promoters is usually more effective than endogenous genes [60].

The results of RNA-seq showed that the abundant stress-regulated genes were significantly downregulated in transgenic lines, including *WRKY*, *ERF*, *DREB*, and *NAC* members (Table 1), which encode transcription factors to target downstream genes and regulate their expression in response to environmental stresses [61–64]. The DEGs were mainly associated with responses to abiotic stress and biotic stimulus (Fig. 7A) and related to pathways of hormone signal transduction, phenylpropanoid biosynthesis, MAPK signaling, and plant-pathogen interaction (Fig. 7B). These pathways have been elucidated to transduce drought and other abiotic signals to stimulate stomatal closure and a series of defense responses [8, 13, 65–71]. It indicates that the negative regulation of the ectopically expressed *ZmLAZ1-3* gene on maize drought tolerance may involve numerous signal transduction pathways. However, the detailed mechanisms need further in-depth research.

## Conclusion

The *ZmLAZ1-3* gene is upregulated in response to drought stress and functions on the plasma membrane and chloroplast to negatively regulate drought tolerance of maize by multiple pathways. It suggests that this gene can be modified by mutation, such as CRISPR/Cas9, to improve maize for drought tolerance.

### Electronic supplementary material

Below is the link to the electronic supplementary material.

Supplementary Material 1

Supplementary Material 2

## Data Availability

The original contributions presented in the study are included in the article/Supplementary Material, further inquiries can be directed to the corresponding authors. The RNA-seq datasets generated during the current study are available in the Sequence Read Archive (SRA) repository with bio-project ID PRJNA1064704, and accessed at https://www.ncbi.nlm.nih.gov/bioproject/PRJNA1064704. The accession numbers of these genes are as follows: *ZmLAZ1-3* (Zm00001d034719), *ZmEF1?* (Zm00001d046449) and *AtActin2* (AT3G18780).
