# Supplementary material for: Maize ZmLAZ1-3 gene negatively regulates drought tolerance in transgenic Arabidopsis
Source: BMC Plant Biol. 2024 Apr 5;24:246. doi: 10.1186/s12870-024-04923-x (PMC10996212; doi:10.1186/s12870-024-04923-x)

**Figure S1** RNA-seq data of *ZmLAZ1-3* in different tissue.

**
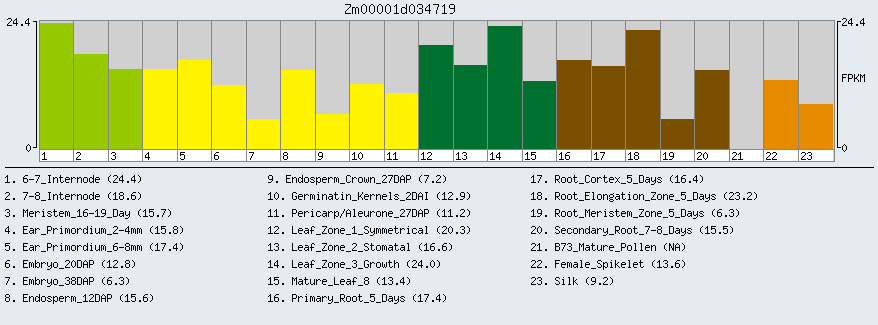
**

**Figure S2** Promoter sequence and cis-affect elements of gene *ZmLAZ1-3*


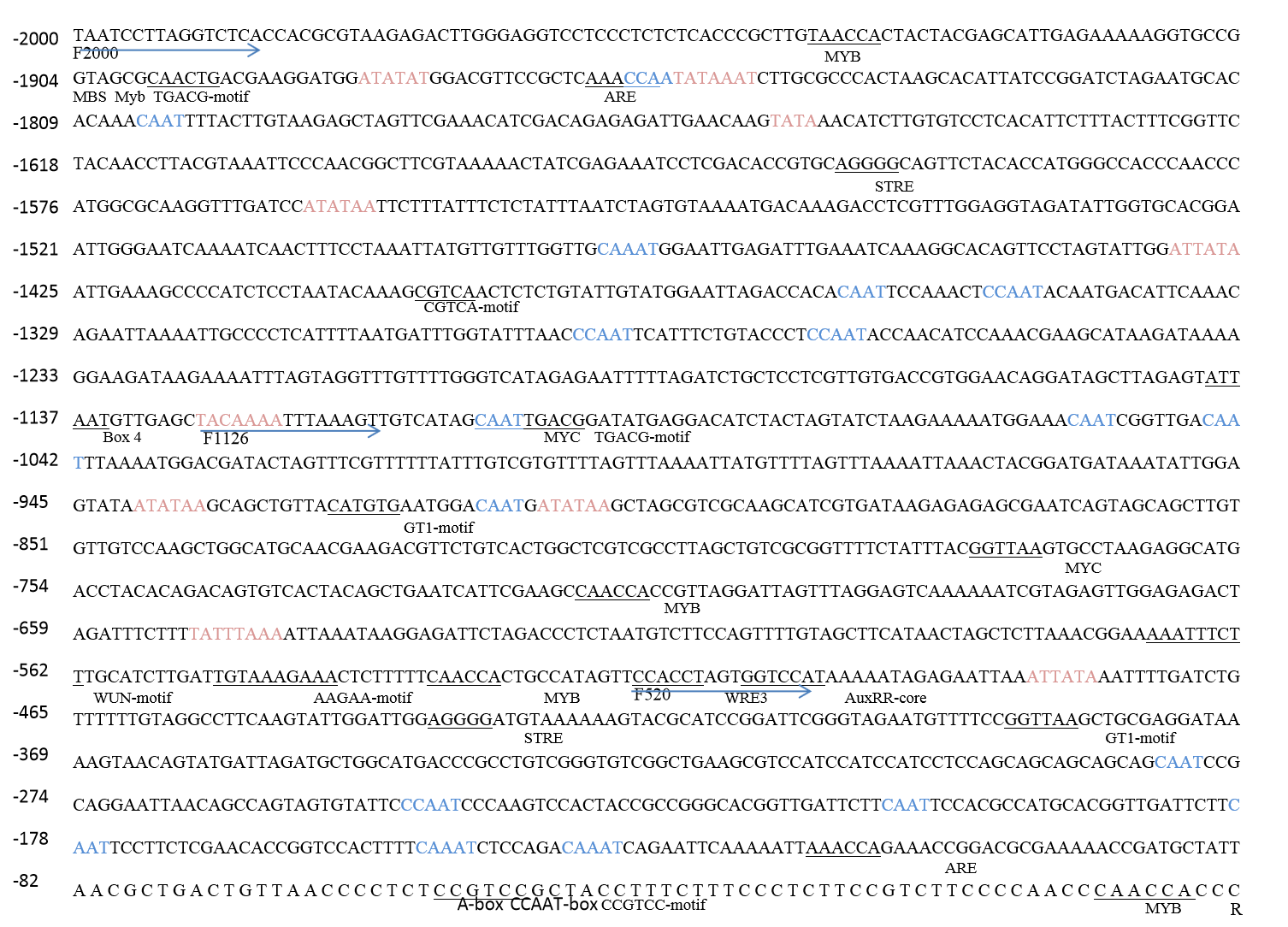

Supplement: Supplementary file 1 — Supplementary Material 1 [file 12870_2024_4923_MOESM1_ESM.docx]
